# Phosphorous Utilization in Microalgae: Physiological Aspects and Applied Implications

**DOI:** 10.3390/plants13152127

**Published:** 2024-08-01

**Authors:** Rosanna Bossa, Melania Di Colandrea, Giovanna Salbitani, Simona Carfagna

**Affiliations:** Department of Biology, University Federico II of Naples, Complesso Universitario MSA, 80126 Naples, Italy; rosanna.bossa@unina.it (R.B.); me.dicolandrea@studenti.unina.it (M.D.C.); simcarfa@unina.it (S.C.)

**Keywords:** eutrophication, microalgae, phosphorus, bioremediation

## Abstract

Phosphorus (P) is a fundamental element for life, playing an integral role in cellular metabolism including energy transfer, nucleic acid synthesis, and membrane structure. This nutrient is critical to the physiological ecology in all photosynthetic organisms including eukaryotic microalgae and cyanobacteria. The review, here presented, delves into the intricate mechanisms governing phosphorus acquisition from the environment, its utilization in plant metabolism, and regulation in these photosynthetic microorganisms. Furthermore, it comprehensively explores the strategies employed by microalgae to cope with phosphorus limitation, such as the activation of high-affinity phosphate transporters and the synthesis of phosphorus storage compounds. On the other hand, the ability to consume abundant phosphate makes microalgae exploitable organisms for environmental remediation processes. The knowledge synthesized in this review contributes to the broader understanding of microalgal physiology, offering insights into the ecological and biotechnological implications of phosphorus assimilation in these microorganisms.

## 1. Introduction

Microalgae represent interesting photosynthetic microorganisms, considered the most primitive on the Earth’s surface [1]. To date, the term “microalgae” refers to both eukaryotic protists and prokaryotic cyanobacteria, that are both unicellular and simple multicellular, with a cellular size ranging from 1 to 400 µm [2,3]. Due to their metabolic variability, microalgae colonized all habitats on Earth, adapting themselves to the most diverse places including extreme environments [2,4,5].

In the last decade, microalgae have aroused considerable interest, stimulating scientists to investigate how to use them as sources of interesting biomolecules or as sustainable solutions for environmental issues [2,3,6,7,8,9]. In fact, they are a valuable source of organic compounds, which can be applied in the sectors of bioenergy, food supplements, agriculture, pharmaceuticals, and cosmetics [10,11,12], but they can be also exploited as bioremediators to correct polluted environments [13,14].

For their growth, microalgae require macronutrients such as nitrogen (N), potassium (K), magnesium (Mg), phosphorus (P), chloride (Cl), and sulfur (S) with trace amounts of vitamins and minerals [15]. In particular, P is an essential macronutrient that is essential for the production of different cellular components and for the metabolic pathways that involve energy transfer and nucleic acid synthesis [16]. Microalgae can adsorb phosphorus by a variety of methods, the two primary ones being extracellular adsorption and intracellular uptake [17]. The main mechanism of extracellular uptake is based on extracellular polymeric substances (EPSs), composed mainly of proteins and carbohydrates, and located on the cell surface of microalgae. In particular, the negatively charged phosphate can bind to the positively charged protein amine groups [18,19].

When exposed to sustained high external P concentrations, microalgae can absorb more phosphorus than is necessary for cell metabolism and growth [17,18,19,20]. The excess phosphorus is then stored in the cells in the form of polyphosphate (polyP) [17,18,19,20]. A low level of accessible P in the environment is frequently encountered too [21]. In response to a P-limited environment, microalgae undergo several biochemical adaptations, including the efficient capture of external P and the redirection of inorganic phosphate (Pi) from intracellular organic-P-containing molecules [22]. Moreover, microalgae and cyanobacteria employ various strategies to conserve P or recycle intracellular P-containing molecules [23,24].

For microalgae cultivation, the optimum content of P in the growth media ranges between 4 mg/L and 500 mg/L, and Table 1 lists the phosphate content in the primary growth media depending on the species. The ability of these microorganisms to assimilate and metabolize phosphorus represents an ability exploited in the treatment of polluted areas. Microalgae are thought to be highly effective microorganisms for wastewater treatment since they can recover biomass and produce products with added value at the same time [25].

This review focuses on the role of phosphorus in microalgae physiology and the biotechnological implications of P utilization in these photosynthetic microorganisms. Here, we also analyze the applicative aspects linked to the use of P by these organisms such as bioremediation or biofertilization.

## 2. Role of Phosphorus in Microalgae

Macronutrients play crucial roles throughout an entire plant organism’s life cycle, contributing to various beneficial activities in metabolism and safeguarding against abiotic and biotic stresses, such as heavy metals, drought, heat, UV radiation, diseases, and insect pest attacks [46,47]. Each macronutrient possesses distinct characteristics, playing specific roles in various metabolic and physiologic processes [21,48].

Phosphorus is a key constituent of nucleic acids (both DNA and RNA), proteins, sugar phosphates, and various metabolites [21,23,48,49]. Additionally, P contributes to the formation of phospholipids, essential components of cellular membranes that provide structure and protection to cells [21,23,48,49]. Phospholipids comprise a significant proportion of cellular phosphorus; it has been estimated that 10–50% of lipids in microalgae are represented by these molecules [50,51].

P is indispensable for carbon fixation and cellular metabolism because it is involved in the production of chemical energy (ATP) and reducing equivalents (NADH, NADPH) during photosynthesis and respiration [21,23,48,49]. Moreover, the essential process of phosphorylation–dephosphorylation forms the foundation for information reception and transfer through signal cascades within the cell [23,48]. The cell’s metabolism, involving energy acquisition, transformation, accumulation, and channeling, is intricately linked to the turnover of a diverse range of P-containing metabolites [23,48].

In addition, P assumes a pivotal role in the physiological ecology of eukaryotic microalgae and cyanobacteria [21,22]. This essential nutrient is actively utilized and transformed by microalgae, contributing to metabolic and biogeochemical dynamics [22]. However, even if these organisms do not need large amounts of P to grow, low algal cell densities are often linked to low concentrations of this element, making P an important growth-limiting factor in natural environments [52]. Furthermore, in microalgae, intracellular P has been identified as crucial for both photosynthetic and physiological characteristics [53,54]. In fact, it has been observed that under P limitation, there is a reduction in chlorophyll biosynthesis and consequently, in photosynthetic rate [55,56].

### 2.1. Phosphorus Uptake

A low level of accessible P in the natural environment is frequently encountered [21,22]. Several biochemical adaptations occur, in response to limited P availability, for microalgae, including the efficient capture of external P and the redirection of Pi from intracellular organic-P-containing molecules [23]. Efficient P uptake involves (1) phosphatases for extracellular degradation of organic P and (2) the synthesis of high-affinity Pi transporters (a decrease in Km) and/or the production of more transporters (an increase in Vmax) to enhance Pi uptake [23,57]. According to Grossman, and Takahashi and Dyhrman et al. [24,58], in response to P deprivation, microalgae synthesize various phosphatases that are upregulated by low phosphate levels. In particular, Dyhrman et al. [24] showed that phosphatases in *Thalassiosira pseudonana* play a key role in hydrolyzing esters from phosphomonoesters, releasing Pi that can be assimilated.

Following 24 h of P starvation, *Chlamydomonas* primarily relies on high-affinity transporters for Pi uptake [58]. Upon recharging with Pi, more than 80% of the total Pi uptake is attributed to low-affinity transporters [59]. Moreover, P starvation amplifies the Vmax for Pi transport by a factor of 10 to 20, enhancing the chances of algal survival [58]. Given the temporal variability of P availability within waters, algae require mechanisms that enable them to sense the concentration of inorganic P (Pi, including PO_4_^3−^, HPO_4_^2−^ and H_2_PO^4−^), both outside and inside the cell, to modulate the activity of hydrolytic transporters and enzymes, and to accumulate excess P and then mobilize it when no longer available [23,49].

The uptake of Pi is also time regulated; in many species it is higher during the day and lower at night, as during the day the photosynthesis increases the cell’s demand for P [49]. For example, a higher rate of P uptake during daytime was highlighted in *Ankistrodesmus convolutus*, *Chlorella vulgaris* [60], and in microalgae grown under the condition of continuous lighting [61].

Genome sequencing has revealed that in microalgae, there is a wide range of inorganic P transporters, located at the plasma membrane, homologous to those of other organisms [21,23]. For example, the model microalgae *Chlamydomonas reinhardtii* have four inorganic P transporters (PTA1-4), similar to the H^+^/P_i_ symporter of higher plants or to the Pho84p and Pho89p transporters also found in *Saccharomyces cerevisiae* [21]. Inorganic P uptake into the cell is performed by active transporters, whose kinetics are generally governed by the Michaelis–Menten equation and are regulated by acclimation to the concentration of inorganic P in the environment, the energy available for the conversion of inorganic P to polyphosphates, and the P demand of the cell [21,49]. Indeed, the plasma membrane P transporters may be high or low affinity, depending on P availability in the environment. In general, high-affinity transporters regulate phosphate uptake under low-P conditions so they are upregulated under low inorganic P availability [49,58]. Experimentally, this determines that when a microalgal culture is placed under conditions of high P availability after exposure to low concentrations of P, it exhibits two-phase kinetics. Specifically, during the first phase, there is an accumulation of P into the cell, generally in the form of polyphosphate. In contrast, the second phase is the exponential growth phase, in which a lower rate of uptake of inorganic P and mobilization of polyphosphate as a source of P is observed [21]. One could argue that the primary role of polyphosphate within the cell is to serve as a P depot [21]; additionally, these molecules are attributed to functions such as storing energy and aiding in the accumulation of crucial metal ions [21]. Microalgal cells typically amass polyphosphate when P is abundant in the surrounding environment [21].

In contrast, low-affinity transporters operate better at high substrate concentrations and thus are upregulated by the surrounding medium, and these polyphosphate reserves are metabolized in times of P scarcity [21]. In other instances, the same transport system operates, but the number of transporters increases under conditions of low P availability [22].

Although microalgae preferentially use Pi, as it can be directly assimilated by cells, under low-Pi conditions they can also use dissolved organic phosphorus (DOP), despite DOP requiring conversion to Pi before being assimilated [62]. The major source of DOP is phosphoesters, which are metabolized through the action of extracellular phosphoesterases. The main types of phosphoesterases are alkaline phosphatases, phosphodiesterases, and 5′ nucleotidases [22].

Alkaline phosphatases hydrolyze Pi from phosphomonoesters, allowing cell utilization. They are commonly found in microalgae and cyanobacteria, and their activity is upregulated primarily by low-P conditions [22]. Indeed, according to Lavrinovičs et al. [63], in *Chlorella vulgaris, Botryococcus braunii, Ankistrodesmus falcatus*, and *Tetradesmus obliquus,* the decrease in P concentration in the medium is correlated with an increase in alkaline phosphatase (AP) activity. The AP has been shown to vary with the taxonomic composition of the microalgae and its activity can be regulated by several biotic and abiotic variables such as temperature, dissolved oxygen concentration, light radiation, and nutrient values [64,65]. In marine phytoplankton, AP activity was usually low at cellular N/P ratios below 14, while higher ratios lead to high AP activity [66,67,68].

Phosphodiesterases, on the other hand, hydrolyze the phosphodiester bond found, for example, in nucleic acids and lipids. The activity of this enzyme is regulated by many factors, specifically, in microalgae and cyanobacteria under P-deficient conditions, where it appears to be involved in the breakdown of phospholipids [22,69]. Last, 5′ nucleotidases hydrolyze phosphate from 5′ nucleotides as ATP or AMP. Like other phosphoesterases, the activity of 5′ nucleotidases is increased by P deficiency [22,70].

### 2.2. Phosphorus Storage

Microalgae exhibit an increased uptake of P under two distinct conditions: when P is reintroduced in the medium after a period of P starvation or deficiency and when the cells are exposed to abundant-P conditions, termed P luxury uptake [23,71]. Despite microalgae typically containing around 1% P in cell dry weight in conditions of low P availability, such as in marine waters, it has been observed that, due to luxury uptake, this value can rise to 4–6% [21,22,72].

**Figure 1:**
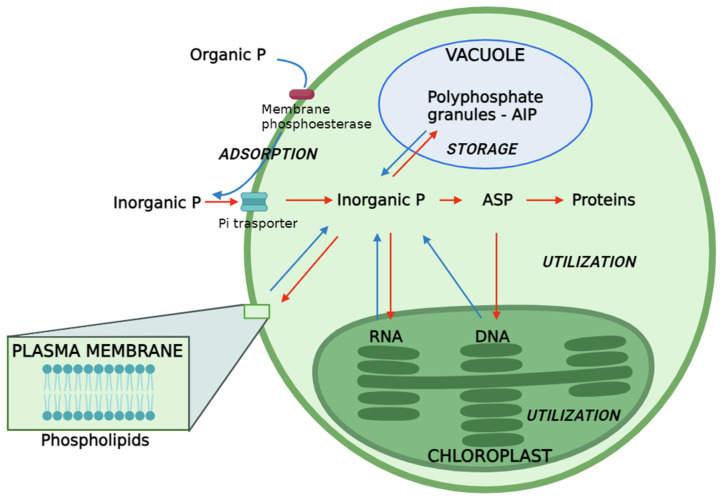
Phosphorus pathways in microalgae. The red arrow indicates the Pi-rich conditions, and the blue arrow indicates the Pi-deficient conditions. ASP: Acid-Soluble Polyphosphate. AIP: Acid-Insoluble Polyphosphate.

As illustrated in Figure 1, the surplus P is accumulated and stored as polyphosphate granules, specifically in the form of Acid-Insoluble Polyphosphate (AIP) within specialized acidocalcisome-like vacuoles [23,73,74,75]. In these vacuoles, polyP forms electron-dense granules that were originally described in yeast as a “volutin” [75,76]. They are colocalized with high concentrations of divalent and monovalent cations [75,77]. This stored P serves for future internal utilization under conditions of Pi deficiency [23,73,74]. In the yeast *Saccharomyces cerevisiae*, as well as in the microalga *Chlamydomonas reinhardtii*, the synthesis of polyphosphates granules is catalyzed by the vacuolar transporter chaperone (VTC) complex and in particular, by the catalytic subunit VTC4. However, in silico analysis based on sequence homologies has shown that polyP synthesis linked to VTC4 activity is conserved in microalgal organisms [75].

Polyphosphate storage is triggered within 1–2 h after P is reintroduced in instances of P starvation and luxury uptake [23,78]. Cliff et al. showed that in several microalgal species (*C. reinhardtii*, *C. vulgaris*, *G. pectorale*, *D. armatus,* and *M. aeruginosa*) grown in low-P conditions, there is a formation of polyphosphate granules and a consequent increase in cellular P content from 1 to 5 h after becoming P replete [75]. Moreover, cell growth does not affect the concomitant production of polyP granules, indicating that their synthesis does not have a high energy cost and that they represent a reserve of P rather than energy [75].

AIP functions as a transfer agent to other cellular P compounds, sustaining algae viability under Pi shortage after being degraded to Pi [23,79]. The polyphosphate granules enable microalgae to undergo several generations without external P until polyphosphate is depleted after prolonged P starvation [23,73,80]. The excess P accumulation beyond immediate needs facilitates P utilization, decoupling P transport from P assimilation to meet the P requirements of microalgae [23,80]. Higher temperatures encourage increased AIP accumulation for P storage purposes and expedite the consumption of ASP [20,23]. Phospholipid formation is also heightened under phosphate-rich culture conditions [23,51].

In general, the concentration of P in microalgal biomass varies greatly from 0.03 to 3% of dry weight [81]. Phosphorus can be found in algae and cyanobacteria in different forms, including phosphomonoester (P-O-C), phosphodiester (C-O-P-O-C), phosphonate (C-P), and polyphosphate (P-O-P-O-P) [22,82,83]. Furthermore, Braun et al. [84] show that in the cyanobacterium *Nodularia spumigena*, following phosphate addition to a P-depleted population, the phosphate concentration decreased in the water as intracellular polyphosphate increased, predominantly in vegetative cells rather than heterocysts. This highlights the distinct roles of these cell types in phosphorus dynamics beyond nitrogen fixation [84]. The relative percentage of each form present depends on the species, but also the growth conditions and the experimental protocol of investigation [22].

Regarding phosphonates, their observation is rare in eukaryotic algae and cyanobacteria. However, 2-aminoethylphosphonate (2-AEP) has been detected in microalgae, including dinoflagellates, such as *Amphidinium carteri*, *Exuviella cassubica*, and *Peridinium trochoidum*, and two species of coccolithophorids, *Coccolithus huxleyi* and *Syracosphaera elongata* [22,85]. This compound is likely to be present in phospholipids and would increase membrane rigidity, as well as act as protection against enzymatic degradation, as the C-P bond is stronger than the ester bond and not attackable by phosphatases [22,86].

### 2.3. Phosphorus Assimilation

Microalgae and cyanobacteria employ strategies to conserve P or recycle intracellular P-containing molecules. These adaptive measures serve to offset their P demand and sustain essential processes, such as ATP synthesis, particularly in response to the absence of extracellular Pi [22,23].

Nucleic acids and lipids are both important phosphorus reservoirs in phosphorus-rich algae [22]. At least 50% of the non-storage P in algae and plants is typically found as RNA within the cells [22,87]. According to the growth rate hypothesis, high concentrations of ribosomes are necessary to sustain rapid growth. Ribosome-rich P content would suggest a positive correlation between growth rate and P content [22,88]. Although the hypothesis may not apply to algae [88], it is consistent with a decrease in ribosomes and rRNA when P is absent. Indeed, Grossman [89] showed cellular RNA decreases in *Chlamydomonas* when Pi is limited and phosphorus-limited cells have fewer ribosomes. Presumably, there is a significant drop in rRNA as protein translation slows down, which permits this phosphorus source to be recycled. The release of Pi can sustain cell viability when chloroplast RNA is degraded by ribonucleases (RNases) [59].

Moreover, phospholipids also are a major P reservoir in photosynthetic organisms, and according to Grossman and Aksoy [59], phosphoinositide might undergo repurposing through the swift breakdown of phospholipids to support vital metabolic processes. Additionally, the membrane composition undergoes a transformation from phospholipids to P-free lipids, such as sulfolipids, predominantly utilizing SO_4_^2−^ instead of Pi [59]. Phosphorus deficiency in *Chlamydomonas* leads to a decrease in phospholipid phosphatidylglycerol (PG) by approximately 50%, with an increase in sulfolipids [90].

In addition, in microalgal cells, polyphosphate, both as Acid-Soluble Polyphosphate (ASP) and Acid-Insoluble Polyphosphate (AIP), undergoes synthesis facilitated by polyphosphate kinase, also known as ATP–polyphosphate phosphotransferases or polyphosphate polymerases. This process involves the transfer of Pi from ATP to elongate the polyphosphate chain, as depicted by the reaction ATP + (phosphate)n ⇄ ADP + (phosphate)n+1 [21,23]. In the presence of light, cellular ASP is actively employed for the synthesis of DNA and proteins during photosynthesis [23,79]. Remarkably, heightened light intensity significantly enhances both the initial accumulation of ASP and its subsequent conversion into proteins and DNA [20,23]. This observation suggests that elevated light intensity positively influences the pathways associated with ASP (Figure 1). The synthesis of RNA and phospholipids predominantly relies on cellular Pi, sourced extracellularly [23,91]. It is noteworthy that the transfer of Pi to RNA is identified as a process inhibited by light [23]. Furthermore, specific organic P species like β-glycerol phosphate and glucose-6-phosphate can pass through the corresponding porins in the outer membrane of cyanobacteria’s cell wall [21].

## 3. Bioavailability of Phosphorus in the Environment

The main source of P for microalgae is the inorganic form, particularly orthophosphate [92,93]. However, it has been demonstrated that several microalgae can also use organic-P-containing molecules to sustain their growth [93,94]. For example, Xing et al. showed that the increase in organic phosphorus in the medium led to greater growth of *Chlorella pyrenoidosa* [93].

In the environment, the main source of P is the phosphate rock, which according to Chowdhury et al. [95], could be exhausted in the next 100 years. Phosphorus is the 11th most abundant element in the Earth’s crust (1200 mg/kg) [96,97]. Despite this, it appears to be poorly available to organisms because, given its high reactivity, it is often bound to chemical compounds that reduce its bioavailability [97]. Indeed, for the terrestrial and aquatic environments, P is an important limiting element.

Most of the phosphorus in aquatic habitats comes from natural slow processes, like the weathering of phosphorus-rich rocks and the deposition of calcium phosphate minerals in marine sediments [22,98]. The charge of inorganic P species present in the environment affects its bioavailability. In particular, inorganic P with a lower charge is more bioavailable than that with a higher charge, e.g., H_2_PO_4_^−^ is more bioavailable than PO_4_^3−^ [21]. Availability may vary over time, follow daily and seasonal variations, or change over the years [49,99]. In particular, the availability of P decreases as temperatures rise during the summer period [100,101]. In water environments, regardless of seasonality and variations in environmental factors, the natural levels of phosphate usually range from 0.005 to 0.05 mg/L [102].

In marine waters, the availability of inorganic P varies greatly from area to area (0.2 nM in the surface waters of the Sargasso Sea, 1–3 μM in the eastern margins of the Pacific and Atlantic, 0.26–5.02 μM in the northern Yellow Sea, 1.51–3.73 μM in the Sea of Marmara) and is generally greater in coastal waters than in the open ocean [49,103,104]. Moreover, P concentration also varies greatly along the water column. In particular, the concentration of inorganic P tends to increase in deep water, due to the uptake of P by phytoplankton and bacterial communities in the euphotic zone [49].

Regarding organic P, its concentration also varies both by area and seasonality. For example, DOP concentration is fairly constant in the Baltic Sea (0.2 μM–0.3 μM) during the spring–summer period [105], while it shows its highest concentration in winter in the Sargasso Sea (6.1 ± 3.5 mmol m^−2^) [106]. In contrast with inorganic P, the concentration of organic P follows an opposite trend, and it is higher at the water surface for two reasons: (1) it does not represent the preferential form of P used by organisms and (2) the pool of organic P is enriched with nucleic acids, free nucleotides, phosphorylated proteins, sugars, and phospholipids released from zooplankton excretions, dead plankton, and the decomposition activity of organic matter by bacteria [49]. The availability of organic P in the oceans may be found mainly in the form of phosphoesters, which contain the C-O-P ester bond, and phosphonates containing the C-P bond. Among these, phosphoesters account for the largest percentage (80–85%) of dissolved organic P. Moreover, some microalgae, such as *Thalassiosira oceanica* CCMP1005 and *Emiliania huxleyi* CCMP374, can be able to absorb polyphosphate with chain lengths reaching up to 130 residues [23,98].

In freshwater, the chemical composition of surface waters is also influenced by atmospheric precipitation, both wet (rain, snow) and dry (aerosol), and their interaction with rocks, vegetation cover, and soil. The concentration of total phosphorus in precipitation averages 33 µg/L, of which, between 20% and 80% is in the soluble form. The largest contributor to the P import in precipitation is from terrigenous aerosols, particularly those from soil erosion, and from vegetation burning. For this reason, the P content in precipitation over the ocean is lower than that over land [107].

Actually, in freshwaters, P is generally found to be a limiting nutrient [108]; despite this, urbanization and agricultural practices have resulted in an enrichment of P concentration at the water surface [105]. In particular, phosphorus inputs to the biosphere have quadrupled in recent decades due to human activities, including the use of P fertilizers for crop production, rock phosphate mining, and intensive land clearance [106,107]. It is well documented that the aquatic content of P is often artificially increased by run-off from agricultural and domestic activities [102]. Globally, it has been estimated that 9.0–14 T/y of anthropogenic P was discharged into freshwater [109,110,111]. Furthermore, Mekonnen and Hoekstra [112] estimate that over half of the total P load was from Asia, followed by Europe (19%), and Latin America and the Caribbean (13%) [112]. The domestic sector accounted for 54% of the total P released into freshwater, agriculture accounted for 38%, and industry accounted for 8% [112]. Within agriculture, cereal production was the largest contributor to the P load (31%), followed by fruits, vegetables, and oil crops, each contributing 15% [112].

## 4. Technological Implications

Phosphorus, being a key macronutrient for plant growth, is massively used for fertilizer production [21]. Despite this, generally less than 20% of the P in fertilizers is actually used by crop species, while the remainder reaches the environment and mainly the hydrosphere [97,113]. Currently, humanity relies on naturally occurring P, extracted from phosphate rock. Extensive phosphate rock deposits are situated in various regions, including Africa (Jordan, Morocco, and the Western Sahara), China, the Middle East, and the USA [114,115]. However, this finite resource remains inaccessible to numerous nations, including the European Union, prompting its classification as a critical resource [115,116]. With rapidly diminishing reserves, the fate of industries dependent on P, particularly agriculture, is uncertain. Consequently, the issue of P scarcity directly impacts food security [115,117].

Also, industrial and especially domestic wastewater may contain high levels of P (Table 2), for example, total P ranging between 5 and 20 mg/L can be found in municipal wastewater, of which, 1–5 mg/L is organic and the remainder is inorganic, commonly in the form of orthophosphoric acid, tripolyphosphate, and pyrophosphate [118,119].

Globally, about ∼380 trillion L/y of wastewater is produced [129]. The excess P present in wastewater can reach the watersheds, causing the phenomenon of eutrophication in natural water [21]. P is one of the crucial nutrients for aquatic biota, serving as a key modulator for phytoplankton growth and algal blooms [130,131,132]. However, the enrichment of water resources with nutrients, especially P and N, leads to a process known as eutrophication, which causes the dense growth of aquatic plant life [130,131,132]. Too high a content of these nutrients in the environment can cause a lower quality of natural waters and adversely affect the entire ecosystem [133]. Microalgae can represent an effective solution for wastewater decontamination; moreover, the produced biomass could be used as a biofertilizer [21,118,134,135].

### 4.1. Microalgae Wastewater Treatment

The biological wastewater treatment technique is able to effectively remove nutrients, recover biomass resources, and realize numerous bioenergy conversions. Industrial and municipal wastewater generally contain high concentrations of pollutants and nutrients, such as P, which can reach concentrations ranging from 1.96 mg/L to 124 mg/L (Table 2).

Microalgae wastewater treatment also has the advantage of having a much lower cost than conventional chemical methods of P removal from wastewater, since wastewater itself contains the nutrients necessary for microalgae growth [118]. The effectiveness of P removal treatment from wastewater by microalgae is influenced by many factors, such as hydraulic retention time, N/P ratio, CO_2_ concentration, and the species of microalgae [119]. Furthermore, although a high Pi concentration is generally favorable for microalgae growth, too high a concentration (>150 mg/L) could be harmful [21,136]. This is a crucial point to consider when utilizing microalgae to decontaminate P-polluted water.

Several studies have shown that the ideal hydraulic retention time, on average, for microalgae to remove P is 6 days [119,129,130,131,132,133,134,135,136,137]. A suitable N/P ratio enhances the P removal efficiency of microalgae and offers a favorable growth environment. The ideal range of N/P ratios for the removal of total P in urban wastewater treated by microalgae using the photobioreactor method is 5–30 [119,138], although the precise value is species-specific and depends on cultivation conditions [139].

Moreover, CO_2_ is essential for microalgal culture growth, as it is the substrate for photosynthesis. Implementing a CO_2_ concentration in wastewater increases the growth rate of microalgae and consequently also the rate of P removal [119]. In general, a concentration of 5–20% CO_2_ promotes nutrient removal, always depending on the microalgae species and environmental parameters, while higher concentrations seem to have a deleterious effect on growth [23,140,141,142,143].

Since each species of microalgae has a different optimum growth characteristic, the choice of microalgae species to be utilized depends on the characteristics of the wastewater to be treated, e.g., pH, C/N ratio, and temperature. One of the most suitable species for wastewater treatment is *Chlorella vulgaris*, which is able to break down the inorganic P content present in synthetic wastewater by 99.2% after 9 days of culture [118]. Moreover, several research studies have shown that using mixed microalgal cultures, in which competition and cooperation are established, increases the rate of P removal in municipal wastewater [119]. Indeed, Khanzada [52] demonstrated that using a co-culture of *Chlorella vulgaris* and *Chlamydomonas reinhardtii*, they were able to decontaminate leachate landfills with 100% removal of P-PO_4_^3^.

Another approach to enhance P removal from wastewater involves the addition of metal ions, such as Mg^2+^, a crucial compound for the functioning of Rubisco activation and thus for the Calvin–Benson cycle [144].

Furthermore, microalgae can also establish symbiotic relationships with bacteria naturally present or added to the wastewater, thereby increasing the degree of wastewater purification [145]. The release, adsorption, and consumption of metabolites as well as the exchange of gas molecules (mainly O_2_ produced by algae and CO_2_ produced by bacteria) are the primary ways in which microalgae and bacteria exhibit their mutually beneficial symbiotic interaction [3,146]. Moreover, microalgae sense phytohormones (3-Indoleacetic acid, IAA), quorum-sensing signal molecules (Acyl-homoserine lactones, AHLs), vitamin B12, and siderophores released by bacteria to increase their resilience to environmental stress [147].

Furthermore, addressing organic P in wastewater could involve utilizing engineered microalgal strains, wherein the stimulation of extracellular alkaline phosphatases enhances biotreatment efficacy [21]. Finally, it is important to note that the accumulation of polyphosphate plays a crucial role in simultaneously incorporating heavy metals (e.g., Al, Ba, Mn, Cd, Co, Cu, Hg, Ni, Pb, Zn), increasing microalgae tolerance to heavy metal toxicity and facilitating the remediation of these contaminations [148,149].

### 4.2. Microalgae as a Biofertilizer

To address the escalating global demand for food, concomitant with population growth, there is an increasing intensification of agriculture [150]. However, the extensive use of chemical fertilizers is causing various issues, including environmental problems like eutrophication and the accumulation of toxic compounds (e.g., heavy metals), as well as potential risks to human health [151].

In response to these challenges, several studies in recent decades have increasingly explored the potential of microalgae as biofertilizers. This section specifically delves into the recycling of P from wastewater treatment to its application in sustainable agriculture.

The green microalgae *Chlorella* [21,72] and *Acutodesmus* [21,152], as well as the cyanobacteria *Anabaena varias, Nostoc muscorum, Aulosira fertissima,* and *Tolypothrix tenuis*, are identified as particularly suitable for biofertilizer production [21,134]. The use of microalgae as biofertilizers offers several advantages. Firstly, the slow release rate of bioavailable P coincides with plant assimilation rates, which reduces the risk of P leaching into groundwater and water eutrophication [21]. Furthermore, microalgae used as biofertilizers provide plant-available P at rates eight times higher than chemical fertilizers, while the actual amount released into the plant is only 3–4.4 times greater, making them more environmentally friendly and sustainable [135]. Additionally, the use of algal biomass eliminates the need for tillage, resulting in time and energy savings [21]. 

Microalgae have immense potential for these applications, yet their use remains primarily restricted to laboratory conditions [153]. Industrial-scale applications have lagged due to significant economic costs associated with large-scale production [153]. Two major challenges are the high cost of artificial growth media and low biomass yield. However, one notable advantage of microalgae-based biofertilizers is that microalgae biomass can be produced not only through conventional methods but also through more sustainable alternatives [153,154]. A promising approach to address these challenges is cultivating microalgae using effluents, which can lower production costs and generate biomass for diverse applications [153]. This approach supports the principles of the circular economy by utilizing reusable resources as a culture medium, making industrial-scale microalgae production more feasible [153].

## 5. Conclusions

This review focused on the crucial role of P as a macronutrient in microalgal cells, in particular, highlighting its role in cell metabolism. The ability of microalgae to uptake and assimilate P from the environment confers to these organisms the capability to heal the environment from P pollution. Beyond its biological role, phosphorus has significant ecological importance; in fact, its excess in aquatic ecosystems can lead to eutrophication, thereby disrupting the trophic chain and causing ecological harm. In addition, the microalgae biomass from bioremediation processes can subsequently have substantial potential for various applications. This review focused on how it can be utilized as a biofertilizer, enriching soil with essential nutrients and promoting sustainable agriculture. However, further research is needed to fully understand phosphorus metabolism within microalgae, useful for refining biotechnological applications to achieve optimal efficiency.

## Figures and Tables

**Table 1:** Comparison of phosphate content in the main microalgae growth media.

Medium	P Form and Concentration	Microalgal Species	References
Allen’s Medium	KH_2_PO_4_ 272.18 mg/L	*Galdieria phlegrea*	[11]
Basal Medium	K_2_HPO_4_ 180 mg/L KH_2_PO_4_ 300 mg/L	*Koliella antarctica*	[5]
Bold’s Basal Medium (BBM)	K_2_HPO_4_ 72 mg/L KH_2_PO_4_ 175 mg/L	*Acutodesmus* sp.	[26]
		*Chlorella protothecoides*	[27]
		*Chlorella vulgaris*	[28]
		*Scenedesmus* sp.	[29]
		*Tetradesmus obliquus*	[30]
Blue Green 11 Medium (BG-11)	K_2_HPO_4_ 40 mg/L	*Anabaena* sp.	[31]
		*Ankistrodesmus falcatus*	[32]
		*Chlorella protothecoides*	[33]
		*Chlorella sorokiniana*	[34]
		*Chlorella vulgaris*	[35]
		*Coelastrella* sp.	[36]
		*Scenedesmus* sp.	[37]
Blue Green 11_0_ Medium (BG-11_0_-modified)	K_2_HPO_4_ 30 mg/L	*Tolypothrix* sp.	[38]
Bristol Medium	K_2_HPO_4_ 74.9 mg/L KH_2_PO_4_ 175.5 mg/L	*Botryococcus braunii*	[39]
Esddekok + Salze Medium (ES)	K_2_HPO_4_ 20 mg/L	*Nostoc muscorum*	[40]
F/2 Medium	NaH_2_PO_4_ 4.32 mg/L Vitamin B12 5015·10^−7^ mg/L	*Nannochloropsis oculata*	[6]
		*Thalassiosira pseudonana*	[41]
7 Medium	K_2_HPO_4_ 0.03 mg/L	*Scenedesmus* sp.	[42]
Tris-Acetate-Phosphate Medium (TAP)	K_2_HPO_4_ 108 mg/L KH_2_PO_4_ 56 mg/L	*Chlamydomonas reinhardtii*	[43]
Watanabe’s Medium	K_2_HPO_4_ 300 mg/L	*Anabaena* sp.	[44]
Zarrouk Medium	K_2_HPO_4_ 500 mg/L	*Spirulina* sp.	[45]

**Table 2:** Total P removal efficiency of microalgae systems in different wastewater treatment.

Wastewater	Microalgal Species	P-Starvation Period (Days)	Treatment Time (Days)	Initial Total P Concentration (mg/L)	Total P Removal Efficiency	References
Municipal	*Desmodesmus communis*	7–14	3	36	>99.9%	[120]
	*Tetradesmus obliquus*	7–14	3	36	>99.9%	[120]
	*Chlorellaprotothecoides*	7–14	10	36	>99.9%	[120]
	*Chlamydomonas* sp.	-	14	-	100%	[121]
	*Spirulina platensis*	-	10	-	88.6%	[122]
Domestic	*Scenedesmus obliquus*	-	10	-	>99.9%	[122]
	*Chlamydomonas reinhardtii*	-	26	1.96	99.15%	[123]
Piggery	*Desmodesmus* sp.	-	14	28.6	81.2%	[124]
Industrial	*Scenedesmus* sp.	-	-	67	88.6%	[125]
	*Desmodesmus* sp.	-	21	-	94–100%	[126]
Textile	*Chlorella* sp.	-	-	17.50	100%	[127]
Sewage water	*Scenedesmus* sp.	5	1	124	86%	[128]
